# Artificial intelligence in ventricular arrhythmias and sudden cardiac death: A guide for clinicians

**DOI:** 10.1016/j.ipej.2025.09.005

**Published:** 2025-09-27

**Authors:** Ibrahim Antoun, Xin Li, Ahmed Abdelrazik, Mahmoud Eldesouky, Kaung Myat Thu, Mokhtar Ibrahim, Harshil Dhutia, Riyaz Somani, G. André Ng

**Affiliations:** a Department of Cardiology, University Hospitals of Leicester NHS Trust, Glenfield Hospital, Leicester, UK; b Department of Cardiovascular Sciences, Clinical Science Wing, University of Leicester, Glenfield Hospital, Leicester, UK; c Leicester British Heart Foundation Centre of Research Excellence, Glenfield Hospital, Groby Road, Leicester, LE3 9QP, UK

**Keywords:** Sudden cardiac death, Artificial intelligence, Machine learning, Ventricular arrhythmias, Personalised medicine

## Abstract

Sudden cardiac death (SCD) from ventricular arrhythmias (VAs) remains a leading cause of mortality worldwide. Traditional risk stratification, primarily based on left ventricular ejection fraction (LVEF) and other coarse metrics, often fails to identify a large subset of patients at risk and frequently leads to unnecessary device implantations. Advances in artificial intelligence (AI) offer new strategies to improve both long-term SCD risk prediction and near-term VAs forecasting. In this review, we discuss how AI algorithms applied to the 12-lead electrocardiogram (ECG) can identify subtle risk markers in conditions such as hypertrophic cardiomyopathy (HCM), arrhythmogenic right ventricular cardiomyopathy (ARVC), and coronary artery disease (CAD), often outperforming conventional risk models. We also explore the integration of AI with cardiac imaging, such as scar quantification on cardiac magnetic resonance (CMR) and fibrosis mapping, to enhance the identification of the arrhythmogenic substrate.

Furthermore, we investigate the application of data from implantable cardioverter-defibrillators (ICDs) and wearable devices to predict ventricular tachycardia (VT) or ventricular fibrillation (VF) events before they occur, thereby advancing care toward real-time prevention. Amid these innovations, we address the medicolegal and ethical implications of AI-driven automated alerts in arrhythmia care, highlighting when clinicians can trust AI predictions. Future directions include multimodal AI fusion to personalize SCD risk assessment, as well as AI-guided VT ablation planning through imaging-based digital heart models. This review provides a comprehensive overview for general medical readers, focusing on peer-reviewed advances globally in the emerging intersection of AI, VAs, and SCD prevention.

## 1 Introduction

Cardiovascular disease is a global issue, and its management can be challenging, especially in the developing world [[1], [2], [3], [4], [5], [6]]. Sudden cardiac death (SCD) due to ventricular arrhythmias (VAs) accounts for 15–20 % of all deaths in industrialised nations and contributes to more years of life lost than most cancers [7,8]. Despite decades of research, accurately identifying individuals remains a challenge. At high risk of SCD, it remains a major clinical challenge. Current guidelines rely heavily on a severely reduced left ventricular ejection fraction (LVEF< 30–35 %) as the primary criterion for primary prevention ICD implantation [9]. While this strategy has saved lives, its limitations are stark: it captures only ∼20–30 % of those who will suffer from SCD [10], meaning the majority of SCD cases occur in patients who fall outside these criteria. Conversely, most patients who do receive prophylactic ICDs never actually use them; fewer than 5 % of ICD recipients receive appropriate life-saving shocks per year [[11], [12], [13]]. This imbalance underscores the need for more sensitive and precise risk stratification tools.

Traditional risk factors and scoring systems often have moderate predictive power at best and can be inconsistent across patient subgroups [14]. Many individuals who suffer from SCD have no previously diagnosed heart condition, and thus would not have triggered any existing risk stratification schema [15]. At the same time, some patients currently receiving ICDs under guideline indications might be spared invasive therapy if we could more accurately determine their true risk of life-threatening arrhythmias.

Artificial intelligence (AI) has emerged as a promising tool to bridge this gap. By leveraging complex algorithms (including machine learning [ML] and deep learning [DL]) on large datasets, AI can uncover subtle patterns and nonlinear relationships in data that elude traditional statistical methods [7]. In the context of VAs and SCD, AI methodologies have been applied to various data sources, including digital ECG signals, imaging modalities (such as cardiac magnetic resonance [CMR] scar imaging), electrophysiologic data from ICDs and wearable sensors, as well as electronic health records and genomic data. AI models are now being developed to improve long-term SCD risk prediction (identifying which cardiomyopathy patients truly need an ICD) and enable near-term prediction of malignant arrhythmia events (alerting patients or physicians hours to days in advance of an impending VAs). Early studies show that these approaches can substantially outperform conventional risk models [14].

In this review, we will examine four core themes at the intersection of AI and arrhythmia care. First, we examine the application of AI to ECG data for SCD risk prediction in conditions such as hypertrophic cardiomyopathy (HCM), arrhythmogenic right ventricular cardiomyopathy (ARVC), and post-myocardial infarction. Next, we discuss AI's role in analysing cardiac imaging (scar and fibrosis) to refine risk stratification and guide therapy. We then review how AI can utilise ICD and wearable device data for predicting VA in the near term, potentially enabling proactive interventions. Finally, we address important liability and trust considerations when deploying automated AI alerts in clinical practice. Additional features include a clinical “pearl” box on when to trust AI for SCD prediction, a schematic diagram of imaging-AI fusion for VT ablation planning, a case vignette illustrating AI-guided decision-making for a borderline ICD case, and a summary table comparing AI-based versus guideline-based risk stratification. Through these discussions, general medical readers will gain a comprehensive understanding of how AI is transforming the field of VAs and SCD, and how to interpret and integrate these advances into practice responsibly.

## 2 ECG-based SCD risk prediction

The 12-lead electrocardiogram (ECG) is a ubiquitous, inexpensive tool that encodes a wealth of information about cardiac electrical activity. Clinicians have long recognized certain ECG abnormalities as markers of elevated arrhythmia risk. For example, QRS prolongation, T-wave alternans, early repolarisation patterns, or deep T-wave inversions in specific conditions. However, conventional ECG criteria and risk scores have limited sensitivity and specificity when used alone for SCD prediction [7]. AI methods, particularly DL, offer a way to harness the full richness of ECG waveforms, identifying subtle patterns or combinations of features that human readers may not easily discern [16].

**AI and the 12-Lead ECG:** DL models can analyse raw ECG signals in their entirety (thousands of data points per lead) to detect prognostic signatures. A recent study by Holmstrom et al. exemplified this approach: researchers trained a convolutional neural network on 12-lead ECGs from patients who later suffered out-of-hospital SCD (versus controls), and achieved an area under the ROC curve (AUROC) of ∼0.89 in internal testing and 0.82 on external validation [7]. This AI-ECG model significantly outperformed a traditional ECG risk score (which had AUROCs ∼0.71–0.74) in distinguishing individuals who would experience SCD from those who would not. Notably, the AI model used only the ECG data itself, without requiring manual measurements of specific intervals or voltages, thereby capturing complex morphologic patterns across leads. Furthermore, another model including 32,294 ECGs from 10,728 demonstrated an AUROC of 0.91–0.95 to predict SCD and unexpected intensive care admissions [17]. Such tools could potentially be used for large-scale screening, for example, by flagging high-risk individuals in the community who have a normal LVEF and no prior diagnosis, so that they can undergo further evaluation. Researchers are also exploring AI-ECG algorithms tailored to specific conditions known to carry SCD risk:

**HCM:** Patients with HCM are prone to lethal arrhythmias despite often having normal or hyperdynamic systolic function. Risk stratification in HCM traditionally uses a combination of clinical factors (extreme wall thickness, family history of SCD, unexplained syncope, non-sustained VT) to estimate annual SCD risk. These methods have modest accuracy, and guidelines can be inconsistent between regions [14]. AI offers an alternative by focusing on the ECG, which in HCM nearly always shows abnormalities even when echocardiographic risk factors are borderline. In one study, ML was used to cluster HCM patients’ ECGs into distinct phenotypes; notably, an ECG pattern characterised by primary T-wave inversions with normal QRS duration was associated with the highest SCD risk profile [18]. This type of unsupervised learning approach can uncover **ECG phenotypes** that correlate with arrhythmic risk markers, potentially refining our understanding of how to interpret HCM ECGs. Supervised DL has also shown promise: convolutional neural networks trained on HCM patient ECGs were reported to achieve ∼85–87 % accuracy in predicting which patients would go on to have SCD events [19]. While these algorithms are still under investigation, they substantially outperform existing clinical risk scores in HCM and could eventually inform ICD decisions in borderline cases.

**ARVC:** ARVC is an inherited disease causing fibrofatty replacement of right ventricular myocardium and VT/VF, often in young people. ARVC diagnosis itself is challenging, requiring a combination of imaging, ECG, and genetic criteria. AI-based ECG analysis can aid in the earlier detection of ARVC. For example, an AI-enhanced ECG algorithm has been shown to identify ARVC patients with expert-level accuracy, successfully distinguishing true ARVC cases from look-alike conditions and asymptomatic gene carriers [20]. Earlier or more accurate diagnosis of ARVC via AI-ECG could indirectly improve SCD prevention by prompting closer monitoring or prophylactic therapy in high-risk individuals. Furthermore, AI might extract risk information from ARVC ECGs beyond the conventional epsilon waves or T-wave inversions in V1–V3; this remains an active area of research.

**Coronary Artery Disease and Others:** In ischemic heart disease, resting ECGs are often abnormal, but AI can integrate these findings in complex ways [21]. Time-series DL models have been trained on ambulatory single-lead ECG data to predict *future* VA. One recent model utilised dynamic ECG features from 24-h Holter recordings to predict VT up to 2 weeks before its occurrence [22]. It correctly predicted 88 % of imminent VTs in a large validation cohort, with external AUCs around 0.91. This suggests that even in patients with coronary disease or heart failure, where the baseline ECG is static, AI can detect subtle temporal changes or precursors (such as variations in ectopic beat patterns or heart rate trends) that flag impending arrhythmias. Additionally, beyond VT/VF, AI-ECG models have been famously used to unmask silent conditions, such as asymptomatic low EF or silent atrial fibrillation [23], which are themselves risk markers for SCD. Identifying a reduced EF from a normal-appearing ECG using AI can find patients who need echocardiography and possibly an ICD before they ever develop symptoms [24].

In summary, the application of AI to ECG analysis is transforming the prediction of SCD risk. The key advantages are scalability and cost-effectiveness: a standard 10-s ECG can be obtained anywhere, including via smartwatches or phone-enabled devices, making AI-ECG screening feasible even in resource-limited settings. AI models can integrate myriad waveform features into a single risk score or alert. Early studies across diverse populations have shown improved accuracy compared to traditional ECG criteria, although clinical implementation will require extensive validation. Nonetheless, the prospect of an “AI ECG” as a cost-effective early warning tool for SCD risk is highly compelling. It could prompt timely preventive measures (like further testing, medication adjustments, or ICD implantation) in patients who would otherwise appear low-risk by conventional evaluation.

## 3 AI and cardiac imaging (scar quantification and fibrosis mapping)

Myocardial scar and fibrosis are well-established substrates for malignant VAs. Scarring from a prior myocardial infarction, or fibrotic replacement in diseases like dilated cardiomyopathy and HCM, provides the electrophysiological milieu for reentrant VT and VF. Clinical studies have shown that the **extent and heterogeneity of scar** on cardiac imaging correlate with SCD risk: for example, a larger volume of late gadolinium enhancement (LGE) on CMR is associated with a higher likelihood of VT in both ischemic and non-ischemic cardiomyopathies, independent of ejection fraction. However, clinicians have lacked robust ways to quantify this risk for individual patients. This is where AI-driven imaging analysis is making a significant impact.

**AI in scar assessment:** Traditional image analysis might measure total scar percentage or core vs. border-zone scar, but AI can mine far more information from imaging data. A landmark study by Popescu et al. used a DL model to analyse CMR scans from patients with ischemic cardiomyopathy and predict their personalised risk of arrhythmic death [8]. The AI model learned features of the scar (such as texture, topology, and spatial distribution) directly from the images and combined them with clinical variables to output a survival curve for arrhythmic SCD over 10 years. It achieved concordance indices of 0.83 (internal validation) and 0.74 (external test) for risk prediction, substantially outperforming standard clinical risk models that rely on LVEF and other variables [8]. Importantly, this model provided *individualised risk trajectories and even an estimate of uncertainty for each prediction.* In practice, such an approach could help identify a patient with, say, an LVEF of 40 % (above the guideline threshold for ICD) but a high-risk scar pattern who might benefit from an ICD – or, conversely, a patient with LVEF 25 % but minimal scar who might avoid an ICD if the predicted risk is low.

**Non-ischemic cardiomyopathy:** Patients with non-ischemic dilated cardiomyopathy (DCM) present a difficult decision area for primary prevention ICDs because trials like DANISH showed no clear mortality benefit, calling into question the use of ejection fraction alone [11]. Fibrosis on MRI has emerged as a key risk marker in this group. AI can integrate fibrosis data with other inputs. A recent study developed a multimodal DL system that combined LGE-MRI, 12-lead ECG, and clinical data to predict VAs in non-ischemic cardiomyopathy patients [25]. The DEEP-RISK model achieved an AUROC of 0.84 with 98 % sensitivity, significantly outperforming models that use any single modality alone [25]. Notably, the CMR-only branch had an AUROC of 0.80 and the ECG-only branch only 0.54, underscoring that while scar imaging is powerful, combining it with electrical and clinical features yields the best prediction. This multimodal AI was able to flag patients with mild-moderate ventricular dysfunction but significant fibrosis who were at high risk – a group that current guidelines might not fully recognise. The study speaks to a broader theme: **integrative AI models** can synthesise data across domains (imaging, ECG, labs) to capture the multidimensional nature of arrhythmic risk.

**HCM:** In HCM, LGE in the myocardium (often patchy mid-wall fibrosis) has been correlated with arrhythmic events. AI can incorporate LGE distribution along with other data to improve risk stratification in HCM beyond the conventional risk calculator. The recent MAARS study introduced a transformer-based AI that analyses **multimodal inputs**, including the full LGE CMR, echocardiography, radiology text reports, and patient clinical data, to forecast SCD in HCM [14]. MAARS achieved an AUC of 0.89 in internal testing and 0.81 in external validation, outperforming contemporary HCM guideline models by a wide margin (AUC improvement of ∼0.25–0.30). This represents a remarkable leap in a field where prior models struggled to achieve an AUC performance of ∼0.60. Furthermore, the MAARS AI demonstrated *fairness* across demographic subgroups (meaning its accuracy held across different ages, sexes, and races), addressing a concern that some clinical risk scores may not generalise well beyond the cohorts from which they were derived.

**Fibrosis mapping and novel imaging:** Beyond standard LGE, AI can leverage other imaging modalities, too. For instance, emerging techniques such as T1 mapping (for diffuse interstitial fibrosis) or Positron emission tomography (PET) (for nerve innervation or inflammation) could inform AI models to refine risk assessments. However, this is still an experimental approach. Radiomics, the extraction of detailed quantitative features from images, is being applied to CT or CMR to characterise scar heterogeneity; ML on these features can predict propensity for VT. For example, radiomic analysis of myocardial scar on CT (for those who cannot undergo CMR) may identify high-risk “texture” patterns associated with arrhythmia. Combining these with AI could one day allow a simple CT scan to yield an arrhythmic risk score.

**Imaging-AI for therapy guidance:** In addition to risk stratification, AI-imaging fusion is beginning to guide therapy, such as catheter ablation of VT. By creating patient-specific 3D models of the heart from imaging, then simulating electrical activation, AI can help locate critical circuits (this will be illustrated later). For instance, so-called digital twin models use LGE-MRI to construct a virtual heart and then run simulations to find reentrant VT channels. In a prospective study, personalised “digital twins” correctly identified ∼81 % of the electrophysiologically defined slow-conduction zones during actual VT ablation procedures [26]. These AI-derived models predicted the locations of abnormal electrograms and circuit isthmuses, aligning closely with invasive mapping findings. Thus, beyond stratifying who needs an ICD, imaging-based AI might also optimise *how* we treat those at risk – by guiding ablation to eliminate the arrhythmogenic substrate preemptively.

In summary, AI applied to cardiac imaging enables th**e quantitative assessment of scars and risk prediction at an individual level**. It moves us from crude metrics like (LVEF <35 %) or (scar present vs absent) to nuanced prognostication using the full richness of the images. Landmark AI models in ischemic and non-ischemic cardiomyopathies have shown significantly improved predictive performance over guidelines. As these tools undergo further validation, they hold promise to refine patient selection for ICDs, targeting them to those who truly benefit – and to identify new candidates currently missed by guidelines (for example, those with preserved LVEF but dangerous scar patterns). Moreover, integrating imaging with other data via AI may yield a comprehensive risk profile for each patient, ushering in an era of highly personalised SCD prevention.

## 4 ICD and wearable data for VT/VF event prediction

While long-term risk models determine *who* should get an ICD or aggressive therapy, there is another dimension to SCD prevention: predicting *when* a VAs might strike soon [27]. Patients with ICDs or those being monitored by wearables generate continuous data streams (intracardiac electrograms, heart rate trends, activity levels). AI algorithms can potentially detect patterns in these data that forewarn an impending arrhythmic event, enabling preventive action (such as medication adjustments or patient alerts to seek medical care). This is an exciting frontier that shifts the paradigm from static risk stratification to dynamic, real-time risk monitoring.

**ICD telemetry and remote monitoring:** Modern ICDs continuously record various physiologic parameters and device data, including intracardiac electrogram (IEGM) morphology, frequency of premature beats, heart rate variability, patient activity as measured by an accelerometer, percentage of pacing, lead impedances, and other relevant data. Researchers have applied ML to large ICD remote monitoring datasets to predict acute arrhythmia events. In one study, Shakibfar et al. used nine variables from remote ICD interrogations of ∼19,900 patients to predict the occurrence of an *electrical storm* (ES, defined as multiple VT/VF episodes within 24 h) within the next day [28]. Their ML model achieved an AUC of 0.80 for predicting imminent electrical storms. Notably, the most important predictive features were the **percentage of ventricular pacing and the patient's daytime physical activity level**. This suggests certain patterns. For example, a high pacing burden (indicating advanced disease or conduction issues) combined with a sudden drop-in daily activity (the patient feeling unwell) can foreshadow an arrhythmic storm. Another study analysed ICD-derived heart rate variability and rhythm data from 788 patients in the SCD-HeFT trial, aiming to predict ICD shocks in the minutes preceding their occurrence [29]. Using ML, they could predict an impending appropriate shock with an AUC of 0.87 as early as 10 s before the arrhythmia, and an AUC of ∼0.81 up to 5 min before [29]. While 10 s is very short, even that warning could hypothetically be used to alert a patient (via vibration or alarm from the device) so they can ensure their safety (such as sitting, lying down, or pulling over if driving) before the shock. A 5-min warning might even allow a transient therapy like overdrive pacing or drug injection by an on-person system in the future. These proofs of concept illustrate that ICDs are not just therapeutic devices but also powerful sensors whose data can be leveraged by AI for predictive purposes.

**Wearable devices and Holter monitors:** Outside of implanted devices, wearable technologies, including Holter monitors, patch recorders, smartwatches, and even wearable cardioverter-defibrillators (WCDs), generate continuous ECG and vital sign data that AI can analyse for patterns of decompensation or impending arrhythmia. The late-breaking study by Fiorina et al. was a milestone, using DL on 24-h Holter ECG recordings from over 78,000 patients to predict *sustained VT* occurring in the subsequent 14 days [22]. In an external validation of ∼59,000 recordings, their model achieved an AUC of 0.91, with ∼79 % sensitivity and 81 % specificity, for predicting which patients would experience a ≥30-s VT in the next two weeks. It correctly identified 88 % of patients who went on to have very fast VTs (≥180 bpm) [22]. This performance is remarkable and suggests that the algorithm discerned subtle warning signs in the ECG hours to days before the overt arrhythmia. Possibilities include detecting increasing frequency of ventricular ectopy, short nonsustained VT runs, changes in T-wave morphology or dispersion, heart rate turbulence, etc., that precede a major arrhythmic event. The authors even envision extending such near-term prediction to consumer wearables in the future, so that high-risk heart patients could be monitored at home and alerted to seek care if their risk signature becomes acute.

Wearable cardioverter-defibrillators (WCD) already provide continuous rhythm surveillance and automatic shock therapy for high-risk patients who are temporarily without an ICD. They have rudimentary algorithms to detect VT/VF, but AI could greatly improve their specificity (reducing false alarms) and even predict arrhythmias before they happen. For example, an AI might advise a patient wearing a WCD to take prophylactic actions or call their doctor if it forecasts high arrhythmic likelihood in the coming hours. Similarly, smartwatches and fitness bands measure heart rate and sometimes ECG; AI can analyse these data for patterns, such as sudden heart rate variability drops, runs of nonsustained tachycardia, or even changes in activity and sleep that often precede health deteriorations. One recent work found that behavioural pattern data from wearables (daily activity trends) were independently associated with VA occurrence [30]. Integrating such data with medical-grade monitoring could enhance prediction.

**Symptom and clinical data integration:** It's worth noting that SCD is not always as sudden as once thought. Studies have reported that over half of patients experience warning symptoms (such as palpitations, chest pain, or syncope) in the days or hours before SCD [31,32]. However, many individuals do not act on these symptoms promptly. AI could play a role by correlating patient-reported symptoms (perhaps collected via a smartphone app) with sensor data to recognise concerning clusters. For instance, an AI system might combine a patient's complaint of dizziness with their wearable showing frequent couplets of PVCs and a blood pressure drop, to conclude that a sustained VT is likely imminent, triggering an alert for immediate evaluation. In a broader sense, the ability to predict near-term risk could extend the “window of prevention,” allowing interventions such as medication adjustments, starting an antiarrhythmic or beta-blocker when risk is high or early deployment of external defibrillators. A conceptual model has been proposed where AI-driven platforms integrate vital signs, ECGs, symptoms, and possibly laboratory tests (such as potassium levels) to forecast cardiac arrest and dispatch emergency services or issue proactive warnings [7].

**Challenges and progress:** These near-term prediction approaches face several challenges. Arrhythmic events are inherently difficult to predict and relatively rare within a given time frame, raising concerns about false positives. False alarms could cause undue anxiety, “alert fatigue,” or unnecessary hospital visits. Ensuring high specificity is as important as sensitivity in these applications. The studies so far show promise. AUCs ∼0.8–0.9 indicate useful discrimination, but prospective trials are needed to demonstrate improved patient outcomes from acting on AI alerts. Another challenge is data integration and timeliness: algorithms must process data in real-time (or near it) and deliver easy-to-understand risk information to clinicians or patients. Workflows must be developed so that, for example, an alert from a patient's device is transmitted to a monitoring centre or physician who can quickly respond.

Nonetheless, the direction is clear: as AI transforms long-term risk stratification, it is simultaneously enabling a new **paradigm of continuous risk monitoring**. In the future, a high-risk patient may leave the hospital not only with an ICD, but also with an AI-driven monitoring app or wearable device that continuously watches for trouble. Combining long-term and short-term predictions could significantly reduce the incidence of SCD. Long–term models identify who needs protection (ICD or medication), and short-term models ensure that even those under protection receive timely intervention before an arrhythmic event proves lethal.

## 5 Liability issues in automated alerts

The integration of AI into clinical decision-making and the implementation of automated patient alerts raise important ethical and legal questions. In traditional care, a physician's judgment is the linchpin of decisions, such as prescribing an ICD or responding to an arrhythmia alarm. However, when an AI algorithm is involved, especially one that might automatically trigger an alert or recommendation, the lines of responsibility can become blurred. **Who is liable if an AI-driven prediction is wrong?** This can manifest in two worrisome scenarios: false negatives (the AI fails to warn of an arrhythmia that then occurs, potentially causing harm) or false positives (the AI triggers unnecessary interventions or anxiety).

One key principle emerging in discussions of medical AI is that these tools should be *assistive*, not autonomous, inpatient care. Clinicians are expected to supervise, interpret, and, when necessary, override AI recommendations. Indeed, patients themselves often expect that **doctors will vet AI outputs** and not simply follow them blindly [33]. In an interview study of patients at risk for SCD, participants expressed that responsibility for final decisions should rest with physicians, who must integrate AI predictions with the patient's context and values [33]. This perspective implies that if an AI suggests “ICD indicated” but the doctor disagrees (or vice versa), the doctor's clinical judgment – and thus liability – still reigns. If a physician were to rely on an AI tool that turned out to be flawed, they could arguably be held liable for not exercising appropriate oversight, similar to how one might be liable for misreading a diagnostic test. Legal experts note that **individual clinicians can be held liable for failing to interpret or verify an AI's output reasonably**, just as they are if they misinterpret a human-generated test result [34].

On the other hand, when AI becomes more autonomous (for example, an algorithm in an implanted device that independently decides to alert or shock a patient), the liability may shift more toward the device manufacturer or software creator. Some argue that *creators of autonomous AI systems should assume liability for harms* when their device is used as intended [35]. In other words, if an FDA-approved AI algorithm in a defibrillator causes a patient injury due to a software error (say, inappropriate shock due to misidentified rhythm), the company could be liable under product liability law, much as if a pacemaker malfunctioned. Legal scholars suggest that manufacturers may need to carry malpractice or error insurance for their AI products [35], and regulatory bodies will likely mandate rigorous validation and post-market surveillance for AI algorithms in high-stakes settings, such as arrhythmia detection.

**False Alarm Fatigue and Patient Harm:** Automated alerts, if too frequent or nonspecific, can also indirectly cause harm. The wearable cardioverter-defibrillator (WCD) experience is instructive: WCDs sometimes issue “LifeVest alarms” for non-sustained arrhythmias or noise, which can cause patients to panic. Inappropriate shocks from WCDs or ICDs due to algorithm misclassification of rhythms have led to trauma (both physical and psychological) and even injuries (falls, burns) in some cases [36]. If an AI algorithm increases its sensitivity to predict events, it may also increase the number of false alarms. Who is responsible if a patient crashes their car because an app erroneously alerts them of a VA, causing panic? These hypothetical scenarios highlight the importance of carefully calibrating AI alert systems. Liability might hinge on whether the AI was reasonably designed and tested to minimise false alerts, and whether the clinician appropriately educated the patient on how to respond to an alert.

**When AI Disagrees with Guidelines:** Another liability consideration is when AI recommendations conflict with standard guidelines. For instance, imagine an AI risk calculator recommends an ICD for a patient who does *not* meet guideline criteria. If the physician follows AI and an adverse outcome from the ICD (like infection or inappropriate shock) occurs, was that deviation from guidelines defensible? Conversely, if the physician ignores the AI and the patient has SCD, could not using an available AI tool be seen as negligence in the future? Currently, the standard of care does not yet formally include AI algorithms, but as they become validated and widely adopted, the standard may evolve. There is even debate about whether *not* using a proven AI tool could one day be considered a breach of duty, if that AI is known to improve the detection of risk [37]. In essence, the medicolegal landscape will need to strike a balance between innovation and responsible practice: clinicians should utilise AI as an aid while maintaining critical oversight.

**Regulatory and Ethical Frameworks:** Regulatory agencies are developing pathways for AI in medical devices, including alert systems. Typically, an AI algorithm intended to influence clinical action is classified as a medical device and requires approval. This vetting process provides some assurance of safety and effectiveness, and in turn might shield clinicians who use an approved tool as intended. However, with “black box” AI, explainability is a concern. If an AI cannot explain *why* it flagged a patient as high-risk, it may be harder to defend its recommendations in court or to gain clinician trust. This is spurring efforts in **explainable AI** where algorithms highlight key factors or provide a rationale for their predictions, which could both improve physician acceptance and provide a clearer chain of accountability.

In summary, automated AI alerts in arrhythmia care introduce a shared responsibility model: developers must ensure their algorithms are thoroughly validated and provide fail-safes; clinicians must exercise judgment and not defer blindly to AI; and healthcare systems must create guidelines for responding to AI alerts. From a liability standpoint, until clearer case law emerges, the prudent approach for clinicians is to treat AI recommendations similar to a consult. Valuable input that must be weighed with all other clinical information. Documenting the rationale for following or not following an AI's suggestion will be important. Ultimately, maintaining the **“human in the loop”** is not just best practice for patient safety, but also the best protection against medicolegal risk when deploying AI for SCD prediction.

## 6 Clinical pearl: when to trust AI in SCD prediction

**Validate the algorithm's provenance:** Trust AI tools that have been trained and validated on populations similar to your patient. Verify if the model has undergone external validation and has published performance results. Be cautious using an algorithm outside its intended setting (such as applying an HCM-specific model to a different cardiomyopathy).

**Use AI as an adjunct, not a replacement:** An AI prediction should complement clinical judgment. If an AI flags a patient as high-risk for SCD contrary to guideline metrics, take it as a cue for deeper evaluation (additional imaging, EP study) rather than an automatic trigger for invasive therapy. Conversely, if AI suggests low risk but the patient has significant conventional risk factors, investigate the discrepancy – there may be unique patient features the model isn't accounting for.

**Look for explainability and consistency:** Prefer AI systems that provide some explanation for their predictions (highlighting which ECG leads or which imaging features influenced the risk score). This transparency can help you determine if the prediction makes clinical sense. If an AI's output is a black box, rely on it only when it aligns with clinical intuition or other evidence. Sudden, large swings in risk estimate without a clear reason should prompt scepticism and possibly a repeat analysis or second opinion.

**Beware of edge Cases:** Understand when an AI might be out of its depth. For example, patients with unusual device data, rare genetic conditions, or poor-quality inputs (noisy ECGs, artefact-laden images). In such cases, the AI's accuracy may drop. If your patient significantly differs from the training cohort (say, an older patient with multiple comorbidities when the model was built on younger patients), be more cautious in trusting the AI's risk estimate.

**Monitor ongoing calibration:** AI models may “drift” or lose calibration over time or when applied to new populations. Encourage the use of AI that comes with calibration information or periodic updates. If possible, participate in registry or monitoring programs that track the AI's performance in real-world use. An AI that was once highly accurate but is now overcalling risk (due to changes in practice patterns or patient demographics) should be recalibrated before you rely on its outputs for critical decisions.

In essence, trust in AI for SCD prediction is earned, not given. The most reliable scenario is when **AI predictions align with clinical evidence** or fill in gaps with additional insight – such as an AI confirming that a borderline patient truly has high-risk features or identifying risk that standard measures miss (like scar-related risk in a preserved ejection fraction patient). Always integrate AI predictions into the broader clinical picture and maintain an open dialogue with patients. Explain when an AI tool is being used and how its recommendation fits into the decision-making process. With these precautions, AI can be a powerful ally in preventing SCD, while the clinician remains the ultimate steward of the patient's care.

**Fig. 1:**
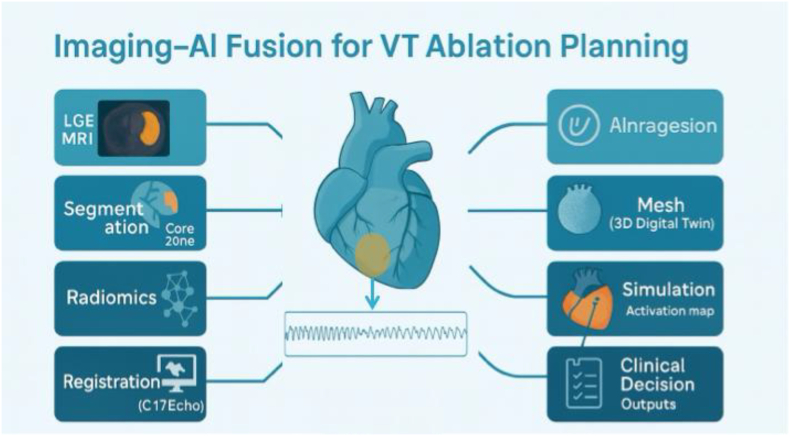
AI fusion for VT ablation planning. Workflow illustrating the integration of imaging and AI for guiding ablation strategies in VT. The process begins with cardiac imaging to delineate structural abnormalities, followed by scar segmentation to identify core and border zones. Extracted features are processed using AI-based analysis, incorporating radiomics and computational modelling. These outputs are integrated into VT ablation planning, including creation of three-dimensional meshes and digital twin simulations, with subsequent generation of activation maps and identification of potential reentry circuits. The final stage supports clinical decision-making, enabling targeted ablation of arrhythmogenic substrate. AI: artificial intelligence; VT: ventricular tachycardia; 3D: three-dimensional. LGE: late gadolinium enhancement. MRI: magnetic resonance imaging.

**Fig. 2:**
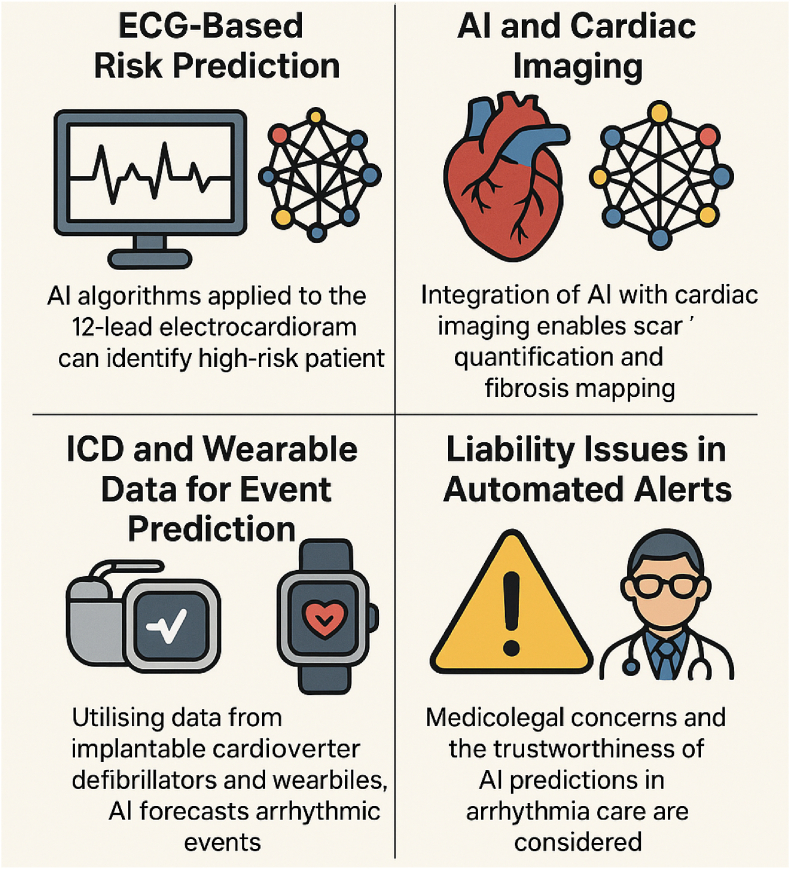
Core domains of AI in VT and SCD. This schematic highlights four principal applications of AI relevant to risk stratification and management. Top left: ECG-based risk prediction, where AI algorithms applied to the 12-lead electrocardiogram can identify high-risk patients beyond traditional criteria. Top right: AI combined with cardiac imaging facilitates the quantification of scars and mapping of fibrosis, thereby enhancing substrate assessment. Bottom left: integration of ICD and wearable device data allows AI models to forecast arrhythmic events and refine event prediction. Bottom right: liability and ethical considerations, addressing medicolegal concerns and the trustworthiness of AI-generated alerts in clinical practice. AI artificial intelligence. ICD: intracardiac defibrillator.

*Imaging-AI fusion concept for VT ablation. Above, a patient-specific digital heart model is created from cardiac MRI (scar tissue is incorporated into the 3D geometry). AI-driven simulation of electrical activity reveals a spiral wave reentrant VT circuit (coloured activation map) anchored to the scar in the right ventricle (blue region). Such* ***digital twin*** *models can predict arrhythmogenic sites and optimal ablation targets. In this example, the virtual model's predictions (*e.g.*, slow conduction zones) correspond to areas with abnormal electrograms found during real catheter mapping* [26]*. This imaging-AI integration enables electrophysiologists to plan a more precise ablation strategy, potentially reducing procedure time and improving long-term success by focusing on patient-specific arrhythmia substrates.* AI fusion for VT ablation planning is illustrated in Fig. 1, and a summary of the role of AI in VA and SCD is presented in Fig. 2.

## 7 Case vignette: AI-predicted SCD risk leading to ICD implantation in a borderline case

**Fig. 3:**
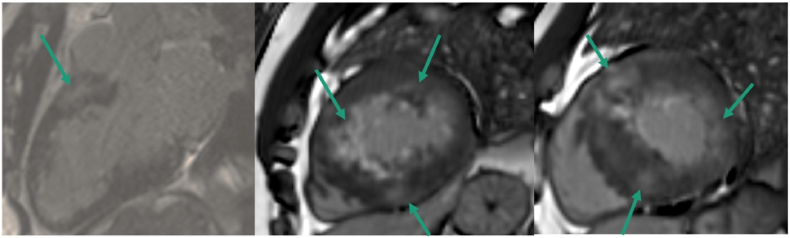
Cardiac magnetic resonance imaging with late gadolinium enhancement in hypertrophic cardiomyopathy. Arrows indicate areas of patchy mid-wall and subepicardial fibrosis in hypertrophied myocardial segments. Such non-ischaemic enhancement patterns represent an arrhythmogenic substrate and are associated with increased risk of ventricular arrhythmias and sudden cardiac death.

A 42-year-old male with HCM presented for routine follow-up. He was asymptomatic, had no family history of SCD, and his conventional risk score using guideline-based calculators estimated a 4 % five-year risk, falling below the recommended threshold for primary prevention ICD implantation. CMR demonstrated patchy late gadolinium enhancement (Fig. 3).

**Fig. 4:**
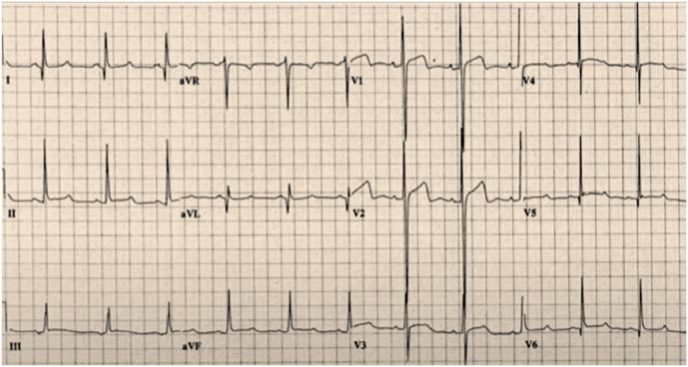
Twelve-lead electrocardiogram illustrating classical features of hypertrophic cardiomyopathy. The tracing demonstrates increased QRS voltages, deep narrow (“dagger-like”) Q waves in the lateral leads (I, aVL, V5-V6), and widespread repolarisation abnormalities, including biphasic T waves.

The patient's 12-lead ECG was analysed by an AI risk model trained on large multicenter datasets of HCM (Fig. 4). The model identified subtle QRS and T-wave abnormalities and integrated these with CMR-derived fibrosis patterns and clinical variables, predicting a significantly higher five-year SCD risk of 8.5 %. After shared decision-making with the patient and considering the AI-augmented risk assessment, an ICD was implanted.

Eighteen months later, the patient experienced a syncopal episode at home. Device interrogation revealed sustained VF terminated by an appropriate ICD shock. The patient recovered fully. This vignette highlights how AI tools may reclassify patients who fall into a “grey zone” under guideline algorithms, demonstrating the potential clinical impact of integrating AI-based risk models into individualised decision-making.

## 8 AI vs. guideline-based SCD risk stratification

To crystallise the differences between emerging AI-driven approaches and conventional guideline strategies for SCD risk assessment, Table 1 compares them across several dimensions and clinical scenarios.

**Table 1:** Comparison of traditional guideline-based SCD risk stratification versus AI-based approaches across different cardiomyopathies. AI methods show improved risk discrimination and individualised prediction but require careful validation and integration with clinical practice.

Aspect	Guideline-Based Stratification	AI-Based Stratification
**Primary risk markers**	Emphasizes coarse metrics (LVEF <35 % for ischemic/DCM; specific risk factors for HCM/ARVC). These markers capture only a fraction of those at risk (e.g., EF criterion covers ∼20–30 % of SCD cases).	Integrates high-dimensional data (ECG waveforms, MRI scar images, etc.) to identify subtle patterns. Can personalize risk beyond standard cut-offs (e.g., detect high-risk scar in a patient with EF 40 % or high-risk ECG in HCM with moderate wall thickness).
**Sensitivity vs. specificity**	Often high specificity but low sensitivity – many who will have SCD are not flagged (∼70 % of SCD in EF >35 % patients). Guidelines aim to avoid over-treating, thus prioritize established high-risk subsets but miss atypical ones.	AI models generally improve sensitivity by utilizing more predictors. For instance, an AI-ECG model detected SCD cases with AUROC ∼0.82 where a conventional ECG score was ∼0.74. Multimodal AI can reclassify “low-risk” guideline patients into higher risk if hidden risk factors present, and vice versa, potentially improving the net reclassification of patients.
**Ischemic CM**	ICD indicated if LVEF ≤35 %. No granular stratification among those above 35 %. Many SCDs occur in patients with intermediate EF; guidelines offer little for them. Scar extent on MRI is recognized as prognostic but not formally in guidelines.	AI analysis of post-MI LGE-MRI predicted 10-year arrhythmic mortality with C-index 0.74–0.83, outperforming models using clinical covariates alone. Patients with preserved EF but high-risk scar patterns can be identified by AI for potential prophylactic ICD, while low-risk patients with low EF could be spared.
**Non-ischemic CM**	EF ≤ 35 % used to justify ICD, but efficacy debated (e.g., DANISH trial showed no clear benefit). No widely adopted risk score; fibrosis presence is noted qualitatively. Treatment decisions often uncertain for EF 36–50 %.	AI multimodal models integrate LGE fibrosis, ECG, and clinical data to predict VT/VF with AUC ∼0.84. They can stratify NICM patients by arrhythmic risk more precisely, identifying those with mild EF reduction but extensive fibrosis who are as high-risk as those with very low EF. AI may guide ICD use more effectively in NICM, improving patient selection.
**HCM**	Uses risk factor scoring (ESC HCM Risk-SCD calculator) combining maximum wall thickness, family history, etc. Moderate performance; some high-risk patients (especially younger ones) may not meet “traditional” markers. LVEF usually normal, so not a discriminator.	AI-driven models (ECG or multimodal) substantially improve prediction. A deep CNN on ECG achieved ∼85 % accuracy for SCD events in HCM. The MAARS multimodal AI (imaging data, electronic health records data) reached AUC 0.81–0.89 and exceeded guideline score performance by 0.25–0.30. AI can thus detect subtle risk in genotype-positive/phenotype-borderline patients, potentially recommending ICDs in cases guidelines would label low-intermediate risk (as in the case vignette).
**ARVC**	Guidelines advise ICD for definite ARVC with extensive disease or prior VT/VF; risk stratification is qualitative (family history, arrhythmic events, right ventricular function) since no universal score. Many ARVC diagnoses are missed or delayed, risking SCD without any intervention. ARVC ventricular arrhythmia calculator.	AI is being used to enhance ARVC diagnosis (AI-ECG can identify ARVC at expert accuracy) and to guide therapy. Experimental AI “digital twins” for ARVC simulate VT based on patient-specific RV scar/genetics, identifying ablation targets and potentially stratifying risk (e.g., which early ARVC patients might need an ICD). As data grows, AI models could provide a more quantitative ARVC risk score, improving on the current subjective approach.
**Transparency and adaptability**	Guideline algorithms are usually transparent (a formula or checklist) and based on clinical trial data. They are, however, infrequently updated and slow to adapt to new risk markers (e.g., genetic information or advanced imaging isn't fully incorporated yet).	Many AI models are “black box” neural networks with limited explainability, which can be a barrier to trust. However, newer explainable AI techniques and user-friendly outputs (risk heatmaps) are emerging. AI models can be retrained periodically as new data arrives (learning from real-world outcomes), offering adaptability. Properly monitored, they can evolve faster than guidelines, though regulatory approval is needed for each iteration.
**Implementation**	Straightforward to implement: based on readily available clinical data. However, by casting a wide net (e.g., everyone with EF <35 % gets ICD), it can lead to many implants with no therapeutic benefit (majority of primary prevention ICDs never fire appropriately). Also, many who will suffer SCD are not intervened upon because they don't meet criteria.	Implementation requires infrastructure: digital tools to obtain AI inputs (ECG digitization, imaging analysis pipelines) and integration into workflows. Some AI algorithms are already deployed (FDA-cleared ECG AI for low EF detection), but for SCD risk they mostly remain in research or limited rollout. If implemented, AI could reduce unnecessary ICDs by better risk discrimination and catch high-risk individuals missed by guidelines, improving overall efficiency of SCD prevention.

AI: Artificial Intelligence; ARVC: Arrhythmogenic Right Ventricular Cardiomyopathy; AUROC: Area Under the Receiver Operating Characteristic curve; AUC: Area Under the Curve; CNN: Convolutional Neural Network; CM: Cardiomyopathy; DCM: Dilated Cardiomyopathy; ECG: Electrocardiogram; EF: Ejection Fraction; ESC: European Society of Cardiology; FDA: Food and Drug Administration; HCM: Hypertrophic Cardiomyopathy; ICD: Implantable Cardioverter-Defibrillator; LGE: Late Gadolinium Enhancement; LVEF: Left Ventricular Ejection Fraction; MAARS: Machine learning Analysis of Arrhythmic Risk in Sarcomeric disease; MI: Myocardial Infarction; MRI: Magnetic Resonance Imaging; NICM: Non-ischemic Cardiomyopathy; RV: Right Ventricle; SCD: Sudden Cardiac Death; VF: Ventricular Fibrillation; VT: Ventricular Tachycardia.

## 9 Conclusion

AI is poised to reshape the landscape of VA and SCD prevention by harnessing vast datasets, including digital ECG footprints, 3D heart images, and continuous wearable signals. AI algorithms can reveal patterns and risk markers with a granularity and scale that are not possible with conventional approaches. The examples reviewed in this article demonstrate that AI can **enhance risk stratification** (identifying high-risk individuals more accurately and earlier), improve the **tailoring of therapies** (such as determining who truly merits an ICD or guiding catheter ablation to critical targets), and enable **dynamic monitoring** for imminent arrhythmic events. Notably, these advances span a range of conditions: inherited diseases like HCM and ARVC, acquired heart disease like post-MI or heart failure, and even general population screening. Early successes include AI models that markedly outperform clinical risk scores in HCM, predict arrhythmic outcomes from cardiac MRI that traditional metrics would overlook, and forecast ventricular tachyarrhythmias days in advance from wearable ECG data [38].

However, translating these innovations into routine care will require surmounting several hurdles. Robust prospective validation is needed – many AI tools have shown promise in retrospective or observational studies. Still, their impact on real-world patient outcomes must be proven (for example, does using an AI risk predictor save more lives or reduce unnecessary ICDs?). Integration into clinical workflows is another challenge: clinicians will need user-friendly software that distils AI predictions into actionable information, ideally accompanied by explanations or confidence levels. There will also need to be education for providers and patients to build trust in AI-assisted care, as well as updated guidelines that incorporate AI tools once they are sufficiently validated. On the technical side, issues of algorithmic bias and generalizability must be addressed. Models should be tested in diverse global populations to ensure they perform well across all demographics, including race, gender, and geography, thereby aligning with the worldwide scope of SCD prevention efforts.

Crucially, the rise of AI does not obviate the fundamentals of sound clinical practice; rather, it augments them. The role of the clinician will evolve to include being an interpreter and steward of AI outputs, verifying that an AI's suggestion makes sense for a particular patient and communicating its meaning effectively. Multidisciplinary collaboration (including cardiology, data science, ethics, and regulatory bodies) will be essential to deploy AI in a way that maximises benefit and minimises harm. As we have discussed, medicolegal frameworks and guidelines will need to catch up to clarify how to use these powerful tools responsibly.

In conclusion, AI's integration into the domain of VAs and SCD is a paradigm shift in the making. It holds the promise of moving from a one-size-fits-all, often reactive approach to a **personalised, proactive strategy**: finding the needle-in-haystack patient who would otherwise be missed, predicting life-threatening arrhythmias before they strike, and optimising therapies to individual patient profiles. The coming years will likely see AI algorithms formally tested in clinical trials (for example, trials where one arm gets AI-guided risk assessment and the other standard care), and their outcomes will inform how quickly these tools become mainstream. If successful, the reward is substantial: fewer sudden deaths, more efficient use of therapies like ICDs, and ultimately a new level of precision in our fight against the long-standing challenge of sudden cardiac death. The fusion of cardiology and AI thus represents not just a technological advance, but a hopeful step toward saving lives that might otherwise be lost without warning.

## Contributorship

GAN, IA: Conceptualization, Writing original manuscript. IA, XL, KMT, ME, AA, MI, HD, GAN and RS: Writing - Review & Editing.

## Funding source

None to declare.

## Declaration of competing interest

The authors declare that they have no known competing financial interests or personal relationships that could have appeared to influence the work reported in this paper.
